# Investigating the Connection between Chronic Periodontitis and Parkinson’s Disease: Findings from a Korean National Cohort Study

**DOI:** 10.3390/biomedicines12040792

**Published:** 2024-04-03

**Authors:** Na-Eun Lee, Dae Myoung Yoo, Kyeong Min Han, Ho Suk Kang, Ji Hee Kim, Joo-Hee Kim, Woo Jin Bang, Hyo Geun Choi, Nan Young Kim, Ha Young Park, Mi Jung Kwon

**Affiliations:** 1 Hallym Data Science Laboratory, Hallym University College of Medicine, Anyang 14068, Republic of Korea; nel2001@hanmail.net (N.-E.L.); ydm1285@naver.com (D.M.Y.); hankm1130@naver.com (K.M.H.); 2 Laboratory of Brain and Cognitive Sciences for Convergence Medicine, Hallym University College of Medicine, Anyang 14068, Republic of Korea; 3 Division of Gastroenterology, Department of Internal Medicine, Hallym University Sacred Heart Hospital, Hallym University College of Medicine, Anyang 14068, Republic of Korea; hskang76@hallym.or.kr; 4 Department of Neurosurgery, Hallym University Sacred Heart Hospital, Hallym University College of Medicine, Anyang 14068, Republic of Korea; kimjihee.ns@gmail.com; 5 Division of Pulmonary, Allergy, and Critical Care Medicine, Department of Medicine, Hallym University Sacred Heart Hospital, Hallym University College of Medicine, Anyang 14068, Republic of Korea; luxjhee@gmail.com; 6 Department of Urology, Hallym University Sacred Heart Hospital, Hallym University College of Medicine, Anyang 14068, Republic of Korea; yybbang@hallym.or.kr; 7 Suseo Seoul E.N.T. Clinic, 10, Bamgogae-ro 1-gil, Gangnam-gu, Seoul 06349, Republic of Korea; mdanalytics@naver.com; 8 Hallym Institute of Translational Genomics and Bioinformatics, Hallym University Medical Center, Anyang 14068, Republic of Korea; honeyny78@gmail.com; 9 Department of Pathology, Busan Paik Hospital, Inje University College of Medicine, Busan 47392, Republic of Korea; hy08.park@gmail.com; 10 Division of Neuropathology, Department of Pathology, Hallym University Sacred Heart Hospital, Hallym University College of Medicine, Anyang 14068, Republic of Korea

**Keywords:** chronic periodontitis, Parkinson’s disease, case-control study nested within a larger cohort, national health screening cohort database

## Abstract

Recent research suggests a potential relevance between chronic periodontitis (CP) and Parkinson’s disease (PD), raising concerns about comorbid PD among elderly CP patients. However, the epidemiologic basis for this association remains unclear. Employing a nested case-control design, this study explored the association between CP and subsequent PD occurrences in Korean adults, leveraging a validated national population-based dataset covering the period from 2002 to 2019. It included 8794 PD patients and 35,176 matched control individuals, established through propensity score matching for age, sex, residential area, and income. Baseline characteristics were compared using standardized differences, and logistic regression was employed to assess the impact of CP histories on PD likelihood while controlling for covariates. We performed a thorough examination of CP events within both 1-year and 2-year intervals preceding the index date, incorporating subgroup analyses. Our analysis revealed no statistically significant association between CP history and PD development overall. However, subgroup analysis revealed a slightly increased likelihood of PD development among CP individuals with a high disease burden (Charlson Comorbidity Index score ≥ 2). In conclusion, although our study did not find a significant overall association between CP history and PD development, the elevated likelihood of PD in subgroups with high disease burden may suggest that comorbidities influence PD probability among certain CP patients. Considering comorbid conditions in PD screening for some individuals with CP may be also important.

## 1. Introduction

Chronic periodontitis (CP) is a prevalent inflammatory condition affecting the supporting structures around teeth, leading to progressive tissue and bone loss [1]. Globally, CP is a significant contributor to tooth loss, impacting approximately 11.2% of the population [2]. In Korea, it accounts for 23.4% of periodontal disease cases, ranking second in outpatient visits, with substantial associated costs [3]. CP incidence rises with age, predominantly affecting males, often associated with lower education levels, income, and rural residence [4]. Influenced by factors such as dental plaque accumulation, specific periodontopathic microbes, and host immune response [5,6], CP can extend beyond oral infection [6], potentially impacting systemic health [7,8], including associations with cardiovascular disease [9,10], diabetes [11], kidney disorders [12], and neurodegenerative diseases [13].

Parkinson’s disease (PD) is a prevalent neurodegenerative disorder characterized by motor and non-motor symptoms, primarily affecting older adults [14]. PD patients commonly experience oral symptoms [15], contributing to oral health issues alongside compromised motor and cognitive control [16,17]. Studies consistently report poor oral health in PD patients, with a high prevalence of CP [17,18,19], suggesting a potential link between PD and CP due to chronic inflammation and dysbiosis [10,13,20]. Given the widespread occurrence of both CP and PD in Korea [3,21], concerns regarding their comorbidity, especially among elderly individuals with CP, have emerged. CP affects 85.5% of individuals aged 60–69 [3], while PD affects approximately 0.4% of those aged 50 and older [21]. Clarifying the potential link between CP and PD holds significant clinical importance [13,22,23], particularly in the absence of preventive measures for PD and considering CP’s global prevalence [2].

The influence of PD on CP is widely accepted [17,18,24], whereas studies linking CP to PD have only recently begun to emerge [25,26,27,28]. However, a limited number of large-scale epidemiological studies and existing meta-analyses examining the risk of PD in CP patients have yielded conflicting results [25,26,27,29,30,31]. Two nationwide epidemiological studies on Korean individuals have investigated the connection between inadequate dental hygiene and the onset of PD, showing an overall weak association between the two conditions [27,31]. While these studies suggest a tendency towards an association between poor oral hygiene and PD risk, these studies did not specifically explore the connection between CP and the incidence of PD [27,31]; the debate persists regarding whether prior CP affects the risk of PD, considering various demographic, socioeconomic, and comorbid factors. Significantly, previous cohort studies have shown inconsistencies in the sizes and demographic data of the study and control groups. For instance, control groups were matched with study groups based solely on age and sex, neglecting socioeconomic status in their analysis [25,26,29], while others did not employ a matching process at all [27,31]. Moreover, the study groups were found to have a higher occurrence of comorbid conditions, including hypertension, diabetes, hyperlipidemia, chronic kidney disease, and stroke [27,29,31], which could potentially introduce confounding factors. As a result, to address the influence of confounding variables, it is recommended to conduct further validation using national population cohort data that ensure demographic balance.

Herein, the primary aim of this research was to explore the connection between CP and the risk of developing PD. To achieve this goal, we conducted a comprehensive analysis using a large, well-matched, validated national cohort dataset obtained from South Korea’s national public healthcare system. We also assessed whether the effect of CP on the likelihood of PD could be affected by individual-specific factors, such as demographics, socioeconomic status, and the presence of other health conditions.

## 2. Materials and Methods

### 2.1. Data Source

The study received ethical clearance from Hallym University’s ethics committee (IRB No. 2019-10-023) and adhered to the Institutional Review Board’s guidelines and regulations. Since the research was based on a secondary analysis of anonymized retrospective data, obtaining written informed consent from participants was not considered necessary.

The main data source utilized in this research was the Korean National Health Insurance Service-Health Screening Cohort (KNHIS-HSC). This cohort comprises anonymized electronic records specifically prepared for research use, thereby safeguarding the privacy of the Korean population by preventing the identification of individuals and this study contains no information that could confirm the personal identities of participants, as previously outlined [32]. South Korea’s National Health Insurance Service covers over 98% of the population with a mandatory national health insurance policy, ensuring wide-ranging representation. In brief, the KNHIS-HSC includes individuals who have taken part in government-sponsored health screening programs. It was established in 2015 by the Korean National Health Insurance Service, using data from national health screening initiatives. Initially, a cohort sample was derived from individuals who underwent health screenings in the years 2002 and 2003, encompassing individuals aged 40 to 79 in 2002. This cohort, tracked until 2019, comprised 514,866 participants from health screenings in 2002 and 2003, representing a 10% random sample of all individuals screened during those years. The Korean National Health Insurance Service is dedicated to the ongoing management and regular updates of this cohort. Given Korea’s population of approximately 55,000,000, this cohort represents about 1% of the total population.

### 2.2. Patient Selection

In this study, diagnostic coding followed the International Classification of Diseases, 10th Revision, Clinical Modification (ICD-10-CM). Initially, from the dataset, individuals aged 40 and older were selected, amounting to a total of 514,866 individuals. These individuals had medical claim codes recorded from 2002 to 2019. Among these, 9437 were identified as PD cases, while 505,429 individuals without PD, and who had no PD diagnosis from 2002 through 2019, served as the control group. To reduce the possibility of bias from pre-existing PD, individuals diagnosed in 2002 and 2003, during a 2-year clearance period (n = 641), along with those lacking fasting blood glucose data (n = 2), were omitted from the study. Similarly, individuals in the control group who were assigned the ICD-10 code G20 even once were removed from the analysis (n = 2082). A propensity score matching approach was employed with a 1:4 ratio to create a control group that closely resembled the PD cases regarding age, sex, region, and income, and, ensuring a more balanced distribution of baseline characteristics. To mitigate selection bias, control participants were arranged through a randomization process, and they were selected sequentially from the top of the list. It was assumed that control participants shared index dates with their corresponding PD cases, leading to each matched pair having the same reference date for comparison (index date).

**Figure 1:**
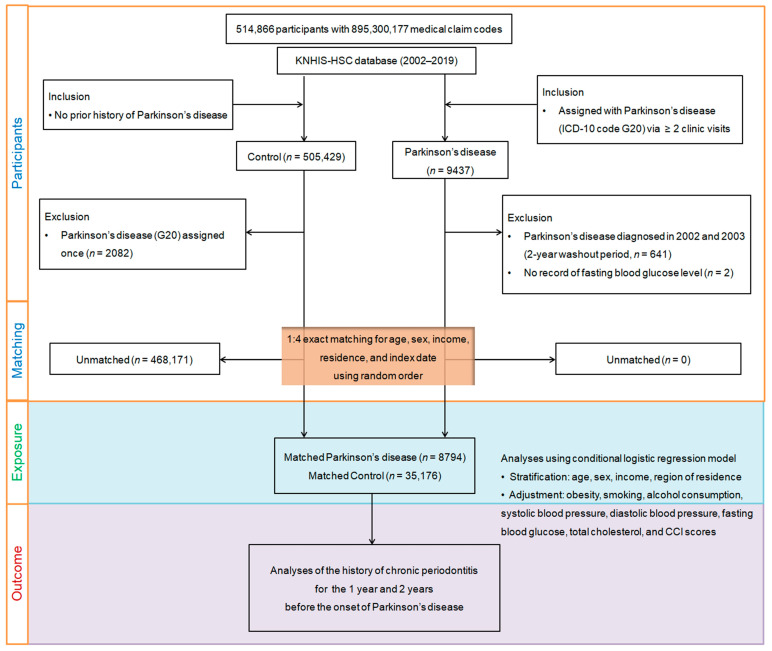
A visual diagram outlining the step-by-step procedure utilized for participant selection in this study. Beginning with the initial pool of 514,866 individuals from the Korean National Health Insurance Service-Health Screening Cohort (KNHIS-HSC) database, a thorough selection procedure resulted in matching 8794 Parkinson’s disease patients with 35,176 control subjects through propensity score matching based on age, gender, income, and residential area.

This strict matching procedure resulted in the removal of 468,171 control members who could not be matched, leaving 8794 individuals with PD successfully paired with 35,176 control participants in a 1:4 ratio for further analysis. Subsequently, the study evaluated both groups for occurrences of CP within 1-year and 2-year intervals before the index date (Figure 1).

### 2.3. Parkinson’s Disease (Outcome)

For PD as the dependent variable, we defined cases based on the ICD-10 code G20 (representing PD), which was confirmed by neurology specialists. Only patients with PD consistently diagnosed with the ICD-10 code G20 during two or more clinical visits were included in the analysis [33].

### 2.4. Chronic Periodontitis (Exposure)

To enhance the analysis’s accuracy and mitigate false-positive cases, only individuals who had undergone treatment for CP by dentists and received a diagnosis confirmed by the specific ICD-10 code (K05.3) were incorporated into the study [34]. The frequency of clinic or hospital visits related to CP treatment was documented on an annual basis. The treatment frequencies for CP were aggregated over a two-year period preceding the index date to provide a comprehensive representation of CP treatment patterns among the participants. This meticulous approach aimed to enhance the robustness of the relationship between CP and the investigated outcomes. 

### 2.5. Covariates

Demographic data and information regarding alcohol drinking, smoking, weight status, and laboratory findings were obtained. The participants were divided into 10 age groups, with each group covering a 5-year range. They were then categorized into five income brackets and classified based on their habitation areas into either urban areas, representing the seven largest cities in Korea, or rural areas, encompassing the remaining regions. Similar categorization was applied to tobacco use (non-smoker, former smoker, current smoker), alcohol consumption (<1 time per week, ≥1 time per week), and body mass index (BMI, kg/m^2^) [35,36], with BMI categories including underweight (<18.5), normal weight (≥18.5 to <23), overweight (≥23 to <25), obese I (≥25 to <30), and obese II (≥30) [37]. Health data such as blood pressure measurements, fasting blood glucose levels, and total cholesterol levels were recorded. The Charlson Comorbidity Index (CCI) is a widely utilized tool for assessing the overall burden of concurrent medical conditions [38], generating a total score ranging from 0 to 29 by considering 17 potential comorbidities, including diabetes, diabetes complications, paraplegia, renal disease, congestive heart failure, acute myocardial infarction, peripheral vascular disease, cerebral vascular accident, dementia, pulmonary disease, connective tissue disorder, peptic ulcer, liver disease, cancer, severe liver disease, metastatic cancer, and human immunodeficiency virus infection [38]. A higher CCI score indicates a greater burden of comorbidities.

### 2.6. Statistical Analyses

To alleviate potential confounding factors and mitigate selection bias, we utilized a propensity score matching technique, aiming to minimize differences between the PD and control groups by calculating propensity scores based on baseline factors such as age, sex, income, and residential area, thus pairing individual PD participants with control participants who demonstrated similar scores [39,40]. 

Continuous variables are presented as means with corresponding standard deviations, while categorical variables are depicted as percentages. To evaluate the equilibrium of matched data between the two groups, we assessed the absolute standardized differences in covariates both pre- and post-matching, considering a covariate adequately balanced if the absolute standardized difference was ≤0.20 [40]. If a covariate still exhibited an absolute standardized difference exceeding 0.20 after matching, additional adjustments were performed using multivariable logistic regression analysis [40]. 

Conditional logistic regression within matched groups was utilized to analyze odds ratio (OR) for subsequent PD relative to CP histories while controlling for age, sex, income, and residence; three models were employed, including a crude model, an adjusted model (model 1) incorporating smoking, alcohol consumption, obesity, and CCI scores, and a further adjusted model (model 2) with additional variables such as fasting blood glucose, total cholesterol, and blood pressure; CP histories were categorized based on treatment frequency (≥1, ≥2, and ≥3 within 1 year, and ≥1 within 2 years), and subgroup analyses were conducted with all covariates considered for each CP history; statistical analysis was conducted using SAS version 9.4 (SAS Institute Inc., Cary, NC, USA), and a two-tailed *p*-value < 0.05 was considered statistically significant.

## 3. Results

### 3.1. Baseline Demographics

Our study encompassed 8794 individuals diagnosed with PD and 35,176 matched control participants, selected from a comprehensive database covering the years 2002 to 2019. Both groups were precisely matched in terms of age, sex, economic status, and geographical location, yielding a standardized difference of 0.00, indicating perfect demographic alignment. Other baseline demographic and clinical features showed standardized differences of ≤0.20, suggesting negligible disparities. However, a slightly higher percentage of individuals in the PD group (69.87%) had a CCI score of 1 or greater, in comparison with the control group (52.16%) (Table 1).

### 3.2. PD Odds Ratios in Relation to CP Histories

We performed a comprehensive analysis of CP occurrences within 1-year and 2-year intervals before the index date. The ORs for developing PD, in both unadjusted and adjusted models (which accounted for demographic characteristics and medical comorbidities), revealed no significant differences between the PD group and controls when considering ≥1 CP history within a 1-year timeframe prior to the index date (Table 2). 

This lack of significant difference between the two groups remained consistent for participants with CP ≥ 2 or CP ≥ 3 histories within 1 year, as well as for those with ≥1 CP history within 2 years (Supplementary Tables S1, Supplementary Table S2, and Supplementary Table S3, respectively). Collectively, our analyses revealed no statistically significant association between the overall history of CP and the onset of PD. 

### 3.3. Subgroup Analysis

**Figure 2:**
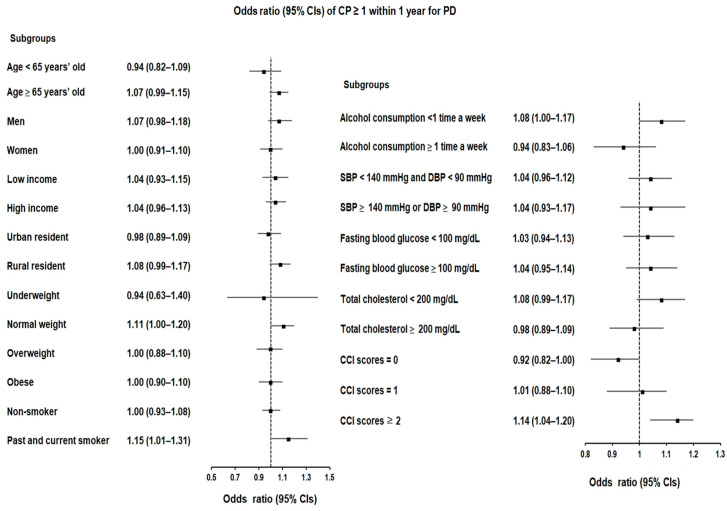
Forest plots depicting the adjusted odds ratio and corresponding 95% confidence intervals (CIs) for demographic, lifestyle, and comorbid factors concerning chronic periodontitis (CP) regarding the occurrence of Parkinson’s disease (PD) when individuals are diagnosed with CP ≥ 1 within 1 year preceding the index date.

**Figure 3:**
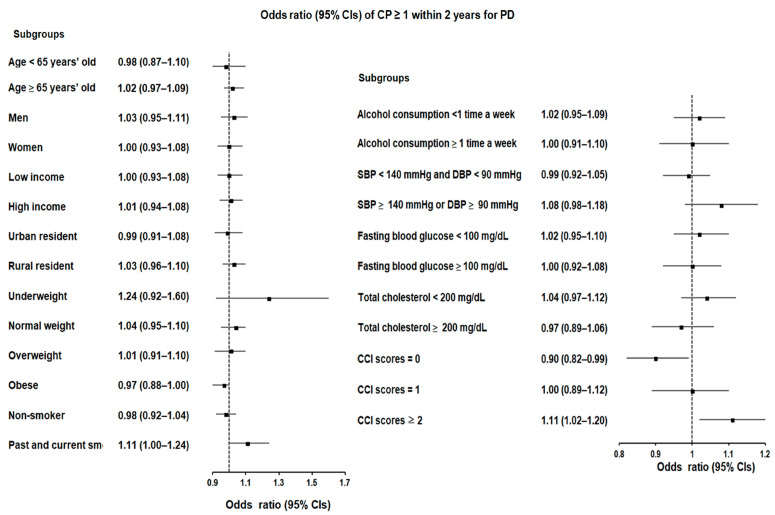
Forest plots depicting the adjusted odds ratio and corresponding 95% confidence intervals (CIs) for demographic, lifestyle, and comorbid factors concerning chronic periodontitis (CP) regarding the occurrence of Parkinson’s disease (PD) when individuals are diagnosed with CP ≥ 1 within 2 years preceding the index date.

We performed a more comprehensive data analysis of the data by categorizing patients based on various parameters, including age, sex, income, residence, weight status, smoking and drinking habits, blood glucose levels, total cholesterol, and CCI scores. Notably, a certain subgroup with a CCI score of ≥2 and a history of CP displayed a slightly elevated likelihood of developing PD. This was evident in cases with at least one CP history within a 1-year timeframe ([OR 1.14; 95% confidence interval (CI) = 1.04–1.26, *p* = 0.008]), cases with at least three CP histories within a 1-year timeframe ([OR 1.20; 95% CI = 1.01–1.42, *p* = 0.034]), and cases with multiple CP occurrences within a 2-year period preceding the index date ([OR 1.11; 95% CI = 1.02–1.20, *p* = 0.012]) (Figure 2 and Figure 3).

On the contrary, additional analyses for CP occurrences of at least one, two, and three instances within 1-year or 2-year intervals revealed inconsistent and temporary associations across different sets. However, in the analysis of CP occurrences within both 1-year and 2-year intervals, a significant association was observed exclusively in the subgroup with a CCI score of ≥2.

## 4. Discussion

The epidemiological foundation for delineating the connection between CP and the onset of PD remains incomplete. This study aimed to evaluate the correlation between CP and the probability of subsequent PD by utilizing a comprehensive, well-matched, and validated national cohort dataset encompassing the adult population of Korea. By leveraging propensity matching for demographic factors, conditional logistic regression analysis that considered comprehensive confounding factors, subgroup analyses, and detailed analyzing medical records spanning a 1-year timeframe (≥1, ≥2, or ≥3 instances of CP histories) and even expanding our analysis to a 2-year timeframe, our findings showed no notable disparities in the ORs for developing PD between the CP and control groups, suggesting that Korean adults with prior CP histories may not exhibit an increased likelihood of developing PD. This study’s meticulous matching of demographic characteristics ensured that the observed outcomes in our study were less likely influenced by confounding factors such as sex, age, economic status, and geographical location. Similarly, our findings are consistent with a prospective cohort study conducted in Sweden involving 15,528 individuals aged 40 years and older [41]. The authors reported no significant association between poor oral health conditions and the risk of PD in either males or females [41]. Additionally, a Mendelian randomization study using genetic data from European ancestry populations also concluded that there was no evidence of genetic predisposition for CP being linked to a higher risk of PD [42]. In a recent meta-analysis involving 4844 participants from six retrospective studies, combined with the aforementioned Mendelian randomization study, it was reaffirmed that CP is not associated with an increased incidence of PD [30]. This suggests a general absence of effect concerning the link between CP and the likelihood of PD, encompassing both clinical presentation and genetic inclination, despite the common occurrence of poor oral hygiene among PD patients [30]. This might imply that the overall presentation of CP could be viewed as a consequence of the poor oral hygiene associated with the clinical symptoms of PD [18,22,24]. Likewise, in a prior Korean cohort study, periodontal diseases were not associated with the onset of new PD cases [27]; however, in unadjusted models, tooth brushing frequency and receiving competent dental care were inversely associated with PD risk [27]. Yet, after accounting for sociodemographic and clinical factors like age, sex, obesity, smoking, alcohol consumption, income, physical activity, and comorbidities, this association became insignificant [27]. This adjusted analysis may suggest that these clinical and environmental factors could potentially influence the association between inadequate oral health and the risk of PD. 

In contrast, two Taiwanese cohort studies indicated that individuals with CP face an elevated risk of developing either PD [26] or Parkinsonism [25]. Another Taiwanese cohort study, conducted by the same authors as one of the previously mentioned studies, suggested that regular periodontal treatment could potentially ameliorate PD-related symptoms, implying that treating CP might have a protective effect on the progression of PD [29]. While prior Korean cohort studies suggested a weak association between poor oral hygiene status and PD risk [27,31], they lacked balanced study and control groups with respect to baseline sociodemographic and health traits. This disparity could lead to heterogeneity resulting from demographic variations and differences in the quality of the study groups [43]. In our study, we utilized nationwide population-based controls matched with propensity scores to attain a more precise equilibrium of baseline characteristics, thereby minimizing study heterogeneity and selection bias. Moreover, we performed multivariate conditional logistic regression analysis to account for potential confounding factors. Using these approaches, we found that although the general CP group may not have a greater risk of PD compared to the non-CP group, a subgroup of the CP cohort with a high burden of medical issues exhibited a slightly increased likelihood of developing PD. Intriguingly, the majority of epidemiologic studies addressing the association between CP and developing PD have been conducted in Asian countries with similar healthcare systems, specifically Taiwan [25,26,29] and Korea [27,31]. Hence, further research is needed to determine whether this relevance may also be observed in other ethnicities.

Interestingly, we noted certain variations in the likelihood of association, particularly among those with a high disease burden. In our study, a slightly elevated propensity for PD was consistently noted over 1 to 2 years among CP patient subgroups with a CCI score of ≥2. This finding may underscore the potential influence of multiple comorbidities on PD development in certain CP patient groups, highlighting the potential importance of considering comorbid conditions when screening for PD in CP patients. Similarly, a previous cohort investigation utilizing the Taiwanese National Health Insurance database revealed that individuals with periodontal inflammatory disease, particularly those with a CCI score of ≥3, exhibited a notably elevated risk of PD following adjustments [26]. Additionally, in a Korean cohort study, the likelihood of incident PD increased progressively from individuals without periodontitis and metabolic syndrome to those with both conditions, with the highest likelihood observed in individuals with both periodontitis and metabolic syndrome [31]. Metabolic syndrome includes various cardiovascular disease-specific risk factors like abdominal obesity, hypertension, diabetes, and hypertriglyceridemia [44]. CP is linked to chronic conditions like chronic kidney disease, cardiovascular disease, diabetes, hyperlipidemia, and malignancy [9,10,11,12,20,45], all of which are considered in CCI scores [38]. Hypertension, diabetes, dyslipidemia, ischemic stroke, hemorrhagic stroke, ischemic heart disease, depression, osteoporosis, and obesity have all been independently linked to a heightened risk of PD in the Korean population [14], collectively accounting for a substantial portion (20.4%) of the population-attributable fraction in PD development [14]. Patients with CP may share common risk factors, such as smoking, sex, ethnicity, oxidative stress, chronic inflammation, and aging, which are also associated with those health conditions [6,9,10,20,22,45,46]. These shared risk factors might contribute to potential common underlying mechanisms, such as systemic metabolic changes accompanied by heightened oxidative stress and ongoing inflammatory conditions [13]. This could render certain susceptible subgroups burdened with comorbidities more vulnerable to PD development. The most recent bioinformatic analyses identified certain differentially expressed genes (*CXCR4*, *CXCL8*, *CD19*, *RPTN*, and *SLC16A9*) in a few datasets of CP and PD [47]. This suggests the possible presence of susceptible individuals for both conditions, although the causal direction between CP and PD cannot be confirmed [47]. Indeed, a gradual increase in hazard ratios for incident PD has been observed with an increasing number of disease components [44], providing possible support for the notion that an increasing disease burden contributes to PD occurrence. 

Our study is subject to several limitations. Firstly, being observational and retrospective, it cannot definitively establish a causal relationship between CP and the development of PD. Our research primarily relies on epidemiological data and does not delve into potential biological mechanisms linking CP and PD, leaving a gap in our understanding of the underlying pathophysiology. Secondly, our study focuses on CP histories within specific time frames (1-year and 2-year periods), which may not capture the potential long-term effects of CP on PD development. Additionally, our findings are based on a specific dataset derived from Korean health insurance system data, which pertains to exposure assessments in Korean adults aged 40 and above. Consequently, generalizing our results to other ethnic populations or different demographic settings may be limited. While our study identified a slightly increased likelihood of PD in certain subgroups, it is important to note that these findings were modest and may warrant further investigation to determine their clinical significance. Moreover, the dataset used, the KNHIS-HSC database, lacked detailed information on factors such as drug use, severity grade of CP and PD, family medical history, personal genetics, or dietary habits. Consequently, our analysis did not account for these missing details. Lastly, despite efforts to adjust for known confounding factors, there is always the possibility of lingering confounding due to undisclosed or unrecorded variables that could influence study outcomes. These limitations may constrain our ability to account for possible undisclosed variables as confounding factors, thereby limiting the scope of our analysis. In addition, evaluating the OR for various CP subtypes (ICD-10 codes K05.30, K05.31, and K05.32) was unattainable, despite the potential intrigue in distinguishing among these three subtypes of periodontitis. This limitation arose from the data accessibility constraints within the KNHIS-HSC database. The ownership of the sample cohort data in KNHIS-HSC lies beyond the authors’ control, requiring researchers to access, analyze, and export the outcomes either by visiting the analysis center or using remote methods.

Nonetheless, this study boasts notable strengths that enhance its robustness. One key strength lies in the utilization of a substantial and representative sample from the Korean adult population, comprising a significant number of participants—8794 individuals with PD and 35,176 well-matched controls—carefully gathered from a validated nationwide healthcare database. This extensive sample instills confidence in the study’s outcomes. Additionally, the meticulous matching of groups based on demographic characteristics, careful attention to the analysis timeframe, and rigorous adjustment for confounding variables further bolster the reliability of the findings. This methodological approach minimizes the impact of potential confounding factors, thereby strengthening the validity of the results. Moreover, the study’s detailed subgroup analysis, incorporating variables such as age, sex, income, residence, and comorbidities, provides a nuanced understanding of the relationship between CP and PD across diverse population segments. The comprehensive nature of the KNHIS-HSC dataset, encompassing complete medical histories from across the entire country, enhances the study’s generalizability and precision. This breadth allows for a more thorough exploration of the potential link between CP and PD. 

## 5. Conclusions

In conclusion, our findings may suggest that within the observed time frames, there is no significant relevance between the occurrence of CP and the development of PD. However, the finding of a heightened probability of PD development in subgroups with high disease burden may suggest that the presence of high comorbidities might contribute to the likelihood of PD in a subset of CP patients. This may highlight the importance of considering comorbid conditions when screening for the possibility of PD in patients with CP. While our study may provide more understanding of the association between CP and PD, particularly emphasizing the role of comorbidities, further research is essential to investigate the long-term implications of CP on PD and to elucidate the biological mechanisms underlying this relationship.

## Figures and Tables

**Table 1:** A summary of the demographic and clinical characteristics of this study.

Characteristics	Total Participants		
	PD	Control	Standardized Difference
Age (y), *n* (%)			0.00
40–64	1676 (19.06)	1676 (19.06)	
65–85+	7118 (80.94)	7118 (80.94)	
Sex, *n* (%)			0.00
Male	4204 (47.81)	16,816 (47.81)	
Female	4590 (52.19)	18,360 (52.19)	
Income, *n* (%)			0.00
1 (lowest)	1624 (18.47)	6496 (18.47)	
2	952 (10.83)	3808 (10.83)	
3	1172 (13.33)	4688 (13.33)	
4	1691 (19.23)	6764 (19.23)	
5 (highest)	3355 (38.15)	13,420 (38.15)	
Region of residence, *n* (%)			0.00
Urban	3326 (37.82)	13,304 (37.82)	
Rural	5468 (62.18)	21,872 (62.18)	
Obesity, *n* (%)			0.01
Underweight	318 (3.62)	1283 (3.65)	
Normal	3098 (35.23)	12,521 (35.60)	
Overweight	2308 (26.25)	9289 (26.41)	
Obese I and Obese II	3070 (34.91)	3070 (34.91)	
Smoking status, *n* (%)			0.09
Nonsmoker	6765 (76.93)	25,888 (73.60)	
Past smoker	1200 (13.65)	5142 (14.62)	
Current smoker	829 (9.43)	4146 (11.79)	
Alcohol consumption, *n* (%)			0.10
<1 time a week	6243 (70.99)	23,295 (66.22)	
≥1 time a week	2551 (29.01)	11,881 (33.78)	
Systolic blood pressure (*n*, %)			0.00
<120 mmHg	2122 (24.13)	8156 (23.19)	
120–139 mmHg	3967 (45.11)	17,428 (49.55)	
≥140 mmHg	2705 (30.76)	9592 (27.27)	
Diastolic blood pressure (*n*, %)			0.11
<80 mmHg	3651 (41.52)	16,604 (47.20)	
80–89 mmHg	3090 (35.14)	12,529 (35.62)	
≥90 mmHg	2053 (23.35)	6043 (17.18)	
Fasting blood glucose (*n*, %)			0.11
<100 mg/dL	4613 (52.46)	20,128 (57.22)	
100–125 mg/dL	2918 (33.18)	11,078 (31.49)	
≥126 mg/dL	1263 (14.36)	3970 (11.29)	
Total cholesterol (*n*, %)			0.05
<200 mg/dL	5169 (58.78)	19,833 (56.38)	
200–239 mg/dL	2501 (28.44)	10,815 (30.75)	
≥240 mg/dL	1124 (12.78)	4528 (12.87)	
CCI score (*n*, %)			0.29
0	2649 (30.12)	16,827 (47.84)	
1	2030 (23.08)	6867 (19.52)	
≥2	4115 (46.79)	11,482 (32.64)	
The number of CP treatments (Mean, SD)			
within 1 year	1.75 (3.98)	1.81 (3.52)	0.01
within 2 years	3.61 (7.05)	3.62 (6.10)	0.01

Abbreviations: PD, Parkinson’s disease; CCI, Charlson Comorbidity Index; CP, chronic periodontitis; SD, standard deviation.

**Table 2:** Crude and adjusted odd ratios of chronic periodontitis (CP) for Parkinson’s disease (PD) when participants are diagnosed with CP ≥ 1 within 1 year before index date.

	N of PD	N of Control	Odd Ratios for PD (95% Confidence Interval)					
	(Exposure/Total, %)	(Exposure/Total, %)	Crude †	*p*	Model 1 †‡	*p*	Model 2 †§	*p*
Total (n = 43,970)								
No CP	7380/8794 (83.9%)	29,689/35,176 (84.4%)	1		1		1	
CP ≥ 1	1414/8794 (16.1%)	5487/35,176 (15.6%)	1.04 (0.97–1.11)	0.265	1.03 (0.97–1.10)	0.323	1.04 (0.98–1.11)	0.228
Age < 65 years old (n = 8380)								
No CP	1374/1676 (82%)	5445/6704 (81.2%)	1		1		1	
CP ≥ 1	302/1676 (18%)	1259/6704 (18.8%)	0.95 (0.83–1.09)	0.474	0.95 (0.83–1.09)	0.461	0.94 (0.82–1.09)	0.416
Age ≥ 65 years old (n = 35,590)								
No CP	6006/7118 (84.4%)	24,244/28,472 (85.2%)	1		1		1	
CP ≥ 1	1112/7118 (15.6%)	4228/28,472 (14.9%)	1.06 (0.99–1.14)	0.103	1.07 (1.00–1.15)	0.061	1.07 (0.99–1.15)	0.086
Men (n = 21,020)								
No CP	3459/4204 (82.3%)	13,986/16,816 (83.2%)	1		1		1	
CP ≥ 1	745/4204 (17.7%)	2830/16,816 (16.8%)	1.06 (0.97–1.16)	0.169	1.08 (0.99–1.18)	0.094	1.07 (0.98–1.18)	0.122
Women (n = 22,950)								
No CP	3921/4590 (85.4%)	15,703/18,360 (85.5%)	1		1		1	
CP ≥ 1	669/4590 (14.6%)	2657/18,360 (14.5%)	1.01 (0.92–1.11)	0.858	1.01 (0.92–1.10)	0.878	1.00 (0.91–1.10)	0.987
Low income (n = 18,740)								
No CP	3186/3748 (85%)	12,812/14,992 (85.5%)	1		1		1	
CP ≥ 1	562/3748 (15%)	2180/14,992 (14.5%)	1.04 (0.94–1.15)	0.479	1.04 (0.94–1.16)	0.397	1.04 (0.93–1.15)	0.508
High income (n = 25,230)								
No CP	4194/5046 (83.1%)	16,877/20,184 (83.6%)	1		1		1	
CP ≥ 1	852/5046 (16.9%)	3307/20,184 (16.4%)	1.04 (0.95–1.13)	0.388	1.04 (0.96–1.14)	0.306	1.04 (0.96–1.13)	0.372
Urban residents (n = 16,630)								
No CP	2741/3326 (82.4%)	10,913/13,304 (82%)	1		1		1	
CP ≥ 1	585/3326 (17.6%)	2391/13,304 (18%)	0.97 (0.88–1.08)	0.608	0.99 (0.89–1.09)	0.819	0.98 (0.89–1.09)	0.745
Rural residents (n = 27,340)								
No CP	4639/5468 (84.8%)	18,776/21,872 (85.8%)	1		1		1	
CP ≥ 1	829/5468 (15.2%)	3096/21,872 (14.2%)	1.08 (1.00–1.18)	0.058	1.09 (1.00–1.18)	0.050 *	1.08 (0.99–1.17)	0.078
Underweight (n = 1617)								
No CP	284/318 (89.3%)	1152/1299 (88.7%)	1		1		1	
CP ≥ 1	34/318 (10.7%)	147/1299 (11.3%)	0.94 (0.63–1.39)	0.752	0.92 (0.62–1.37)	0.677	0.94 (0.63–1.40)	0.753
Normal weight (n = 15,612)								
No CP	2595/3098 (83.8%)	10,655/12,514 (85.1%)	1		1		1	
CP ≥ 1	503/3098 (16.2%)	1859/12,514 (14.9%)	1.11 (1.00–1.24)	0.055	1.12 (1.00–1.25)	0.042 *	1.11 (1.00–1.24)	0.056
Overweight (n = 11,480)								
No CP	1930/2308 (83.6%)	7652/9172 (83.4%)	1		1		1	
CP ≥ 1	378/2308 (16.4%)	1520/9172 (16.6%)	0.99 (0.87–1.12)	0.823	1.00 (0.88–1.13)	0.953	1.00 (0.88–1.13)	0.981
Obese (n = 15,261)								
No CP	2571/3070 (83.8%)	10,230/12,191 (83.9%)	1		1		1	
CP ≥ 1	499/3070 (16.3%)	1961/12,191 (16.1%)	1.01 (0.91–1.13)	0.820	1.02 (0.92–1.14)	0.708	1.00 (0.90–1.12)	0.965
Non-smoker (n = 32,525)								
No CP	5723/6765 (84.6%)	21,834/25,760 (84.8%)	1		1		1	
CP ≥ 1	1042/6765 (15.4%)	3926/25,760 (15.2%)	1.01 (0.94–1.09)	0.741	1.01 (0.93–1.08)	0.877	1.00 (0.93–1.08)	0.988
Past smoker and current smoker (n = 11,445)								
No CP	1657/2029 (81.7%)	7855/9416 (83.4%)	1		1		1	
CP ≥ 1	372/2029 (18.3%)	1561/9416 (16.6%)	1.13 (1.00–1.28)	0.056	1.15 (1.02–1.31)	0.027 *	1.15 (1.01–1.31)	0.031 *
Alcohol consumption < 1 time a week (n = 29,626)								
No CP	1657/2029 (81.7%)	7855/9416 (83.4%)	1		1		1	
CP ≥ 1	372/2029 (18.3%)	1561/9416 (16.6%)	1.10 (1.02–1.18)	0.018 *	1.09 (1.01–1.18)	0.026 *	1.08 (1.00–1.17)	0.044 *
Alcohol consumption ≥ 1 time a week (n = 14,344)								
No CP	2141/2551 (83.9%)	9785/11,793 (83%)	1		1		1	
CP ≥ 1	410/2551 (16.1%)	2008/11,793 (17%)	0.93 (0.83–1.05)	0.243	0.95 (0.84–1.06)	0.362	0.94 (0.83–1.06)	0.292
SBP < 140 mmHg and DBP < 90 mmHg (n = 30,124)								
No CP	4728/5669 (83.4%)	20,524/24,455 (83.9%)	1		1		1	
CP ≥ 1	941/5669 (16.6%)	3931/24,455 (16.1%)	1.04 (0.96–1.12)	0.330	1.04 (0.97–1.13)	0.272	1.04 (0.96–1.12)	0.361
SBP ≥ 140 mmHg or DBP ≥ 90 mmHg (n = 13,846)								
No CP	2652/3125 (84.9%)	9165/10,721 (85.5%)	1		1		1	
CP ≥ 1	473/3125 (15.1%)	1556/10,721 (14.5%)	1.05 (0.94–1.17)	0.387	1.05 (0.94–1.17)	0.405	1.04 (0.93–1.17)	0.460
Fasting blood glucose < 100 mg/dL (n = 24,375)								
No CP	3880/4613 (84.1%)	16,680/19,762 (84.4%)	1		1		1	
CP ≥ 1	733/4613 (15.9%)	3082/19,762 (15.6%)	1.02 (0.94–1.12)	0.619	1.03 (0.94–1.13)	0.499	1.03 (0.94–1.13)	0.476
Fasting blood glucose ≥ 100 mg/dL (n = 19,595)								
No CP	3500/4181 (83.7%)	13,009/15,414 (84.4%)	1		1		1	
CP ≥ 1	681/4181 (16.3%)	2405/15,414 (15.6%)	1.05 (0.96–1.15)	0.281	1.06 (0.96–1.16)	0.238	1.04 (0.95–1.14)	0.414
Total cholesterol < 200 mg/dL (n = 25,115)								
No CP	4316/5169 (83.5%)	16,848/19,946 (84.5%)	1		1		1	
CP ≥ 1	853/5169 (16.5%)	3098/19,946 (15.5%)	1.07 (0.99–1.17)	0.088	1.08 (0.99–1.17)	0.079	1.08 (0.99–1.17)	0.091
Total cholesterol ≥ 200 mg/dL (n = 18,855)								
No CP	3064/3625 (84.5%)	12,841/15,230 (84.3%)	1		1		1	
CP ≥ 1	561/3625 (15.5%)	2389/15,230 (15.7%)	0.98 (0.89–1.09)	0.755	1.00 (0.90–1.10)	0.960	0.98 (0.89–1.09)	0.741
CCI scores = 0 (n = 19,476)								
No CP	2245/2649 (84.8%)	14,105/16,824 (83.8%)	1		1		1	
CP ≥ 1	404/2649 (15.3%)	2719/16,824 (16.2%)	0.93 (0.83–1.05)	0.235	0.92 (0.82–1.03)	0.137	0.92 (0.82–1.03)	0.131
CCI score = 1 (n = 8885)								
No CP	1709/2030 (84.2%)	5796/6855 (84.6%)	1		1		1	
CP ≥ 1	321/2030 (15.8%)	1059/6855 (15.5%)	1.03 (0.90–1.18)	0.689	1.01 (0.88–1.16)	0.874	1.01 (0.88–1.16)	0.874
CCI score ≥ 2 (n = 15,612)								
No CP	3426/4115 (83.3%)	9788/11,497 (85.1%)	1		1		1	
CP ≥ 1	689/4115 (16.7%)	1709/11,497 (14.9%)	1.15 (1.05–1.27)	0.004 *	1.14 (1.04–1.26)	0.008 *	1.14 (1.04–1.26)	0.008 *

Abbreviations: CP, chronic periodontitis; PD, Parkinson’s disease; SBP, systolic blood pressure; DBP, diastolic blood pressure; CCI, Charlson Comorbidity Index. * Conditional or unconditional logistic regression analysis, Significance at *p* < 0.05. † Stratified model for age, sex, income, and region of residence. ‡ Model 1 was adjusted for smoking, alcohol consumption, obesity and CCI scores. § Model 2 was adjusted for model 1 plus total cholesterol, systolic blood pressure, diastolic blood pressure, and fasting blood glucose.

## Data Availability

All data are available from the database of National Health Insurance Sharing Service (NHISS) https://nhiss.nhis.or.kr/ (accessed on 1 September 2023). NHISS allows access to all of this data for any researcher who promises to follow the research ethics at some processing charge. If you want to access the data of this article, you can download it from the website after promising to follow the research ethics.

## Supplementary Materials

The following supporting information can be downloaded at: https://www.mdpi.com/article/10.3390/biomedicines12040792/s1, Table S1: Crude and adjusted odd ratios of chronic periodontitis (CP) for Parkinson’s disease (PD) when participants are diagnosed with CP ≥ 2 within 1 year before index date; Table S2: Crude and adjusted odd ratios of chronic periodontitis (CP) for Parkinson’s disease (PD) when participants are diagnosed with CP ≥ 3 within 1 year before index date; Table S3: Crude and adjusted odd ratios of chronic periodontitis (CP) for Parkinson’s disease (PD) when participants are diagnosed with CP ≥ 1 within 2 years before index date.
