# Supplementary material for: Investigating the Connection between Chronic Periodontitis and Parkinson’s Disease: Findings from a Korean National Cohort Study
Source: Biomedicines. 2024 Apr 3;12(4):792. doi: 10.3390/biomedicines12040792 (PMC11048377; doi:10.3390/biomedicines12040792)
Supplement: Supplementary file 1 [file biomedicines-12-00792-s001.zip › biomedicines-2923541-supplementary.pdf]

Supplementary Table S1. Crude and adjusted odd ratios of chronic periodontitis (CP) for Parkinson's disease (PD) when participants are diagnosed with CP ≥ 2 within 1 year before index date.

	N of AD (exposure/total, %)	N of Control (exposure/total, %)	Odd ratios for PD (95% confidence interval)					
			Crude†	*p*	Model 1†‡	*p*	Model 2†§	*p*
Total (n = 43,970)								
No CP	8109/8794 (92.2%)	32,416/35,176 (92.2%)	1		1		1	
CP ≥ 2	685/8794 (7.8%)	2760/35,176 (7.9%)	0.99 (0.91–1.08)	0.858	0.99 (0.90–1.08)	0.782	1.00 (0.91–1.09)	0.937
Age < 65 years old (n = 8380)								
No CP	1549/1676 (92.4%)	6087/6704 (90.8%)	1		1		1	
CP ≥ 2	127/1676 (7.6%)	617/6704 (9.2%)	0.81 (0.66–0.99)	0.037*	0.81 (0.66–0.99)	0.039*	0.81 (0.66–0.99)	0.037*
Age ≥ 65 years old (n = 35,590)								
No CP	6560/7118 (92.2%)	26,329/28,472 (92.5%)	1		1		1	
CP ≥ 2	558/7118 (7.8%)	2143/28,472 (7.5%)	1.05 (0.95–1.15)	0.368	1.06 (0.96–1.16)	0.273	1.05 (0.95–1.16)	0.345
Men (n = 21,020)								
No CP	3834/4204 (91.2%)	15,352/16,816 (91.3%)	1		1		1	
CP ≥ 2	370/4204 (8.8%)	1464/16,816 (8.7%)	1.01 (0.90–1.14)	0.845	1.02 (0.91–1.16)	0.696	1.02 (0.91–1.16)	0.704
Women (n = 22,950)								
No CP	4275/4590 (93.1%)	17,064/18,360 (92.9%)	1		1		1	
CP ≥ 2	315/4590 (6.9%)	1296/18,360 (7.1%))	0.97 (0.85–1.10)	0.645	0.98 (0.86–1.11)	0.707	0.96 (0.84–1.09)	0.544
Low income (n = 18,740)								
No CP	3495/3748 (93.3%)	13,906/14,992 (92.8%)	1		1		1	
CP ≥ 2	253/3748 (6.8%)	1086/14,992 (7.2%)	0.93 (0.80–1.07)	0.294	0.94 (0.81–1.08)	0.363	0.94 (0.81–1.08)	0.377
High income (n = 25,230)								
No CP	4614/5046 (91.4%)	18,510/20,184 (91.7%)	1		1		1	
CP ≥ 2	432/5046 (8.6%)	1674/20,184 (8.3%)	1.04 (0.93–1.16)	0.535	1.04 (0.93–1.17)	0.451	1.03 (0.92–1.15)	0.608
Urban residents (n = 16,630)								
No CP	3035/3326 (91.3%)	12,058/13,304 (90.6%)	1		1		1	
CP ≥ 2	291/3326 (8.8%)	1246/13,304 (9.4%)	0.93 (0.81–1.06)	0.272	0.94 (0.82–1.07)	0.345	0.93 (0.81–1.07)	0.309
Rural residents (n = 27,340)								
No CP	5074/5468 (92.8%)	20,358/21,872 (93.1%)	1		1		1	
CP ≥ 2	394/5468 (7.2%)	1514/21,872 (6.9%)	1.04 (0.93–1.17)	0.457	1.05 (0.94–1.18)	0.390	1.04 (0.93–1.17)	0.488
Underweight (n = 1617)								
No CP	301/318 (94.7%)	1229/1299 (94.6%)	1		1		1	
CP ≥ 2	17/318 (5.4%)	70/1299 (5.4%)	0.99 (0.58–1.71)	0.976	1.00 (0.58–1.74)	0.990	1.04 (0.60–1.82)	0.887
Normal weight (n = 15,612)								
No CP	2838/3098 (91.6%)	11,579/12,514 (92.5%)	1		1		1	
CP ≥ 2	260/3098 (8.4%)	935/12,514 (7.5%)	1.13 (0.98–1.31)	0.085	1.14 (0.99–1.32)	0.069	1.13 (0.98–1.31)	0.091
Overweight (n = 11,480)								
No CP	2135/2308 (92.5%)	8413/9172 (91.7%)	1		1		1	
CP ≥ 2	173/2308 (7.5%)	759/9172 (8.3%)	0.90 (0.76–1.07)	0.221	0.90 (0.76–1.07)	0.253	0.91 (0.77–1.09)	0.309
Obese (n = 15,261)								
No CP	2835/3070 (92.4%)	11,195/12,191 (91.8%)	1		1		1	
CP ≥ 2	235/3070 (7.7%)	996/12,191 (8.2%)	0.93 (0.80–1.08)	0.349	0.94 (0.81–1.10)	0.447	0.92 (0.79–1.07)	0.281
Non-smoker (n = 32,525)								
No CP	6267/6765 (92.6%)	23,827/25,760 (92.5%)	1		1		1	
CP ≥ 2	498/6765 (7.4%)	1933/25,760 (7.5%)	0.98 (0.88–1.09)	0.693	0.98 (0.88–1.08)	0.647	0.97 (0.87–1.07)	0.520
Past smoker and current smoker (n = 11,445)								
No CP	1842/2029 (90.8%)	8589/9416 (91.2%)	1		1		1	
CP ≥ 2	187/2029 (9.2%)	827/9416 (8.8%)	1.05 (0.89–1.25)	0.533	1.07 (0.91–1.27)	0.399	1.07 (0.90–1.27)	0.433
Alcohol consumption < 1 time a week (n = 29,626)								
No CP	5756/6243 (92.2%)	21,617/23,383 (92.5%)	1		1		1	

CP ≥ 2	487/6243 (7.8%)	1766/23,383 (7.6%)	1.04 (0.93–1.15)	0.507	1.03 (0.93–1.15)	0.554	1.02 (0.92–1.14)	0.660
Alcohol consumption ≥ 1 time a week (n = 14,344)								
No CP	2353/2551 (92.2%)	10,799/11,793 (91.6%)	1		1		1	
CP ≥ 2	198/2551 (7.8%)	994/11,793 (8.4%)	0.91 (0.78–1.07)	0.269	0.93 (0.79–1.09)	0.384	0.93 (0.79–1.09)	0.371
SBP < 140 mmHg and DBP < 90 mmHg (n = 30,124)								
No CP	5216/5669 (92%)	22,452/24,455 (91.8%)	1		1		1	
CP ≥ 2	453/5669 (8%)	2003/24,455 (8.2%)	0.97 (0.88–1.08)	0.623	0.99 (0.89–1.10)	0.789	0.98 (0.88–1.09)	0.730
SBP ≥ 140 mmHg or DBP ≥ 90 mmHg (n = 13,846)								
No CP	2893/3125 (92.6%)	9964/10,721 (92.9%)	1		1		1	
CP ≥ 2	232/3125 (7.4%)	757/10,721 (7.1%)	1.06 (0.91–1.23)	0.488	1.05 (0.90–1.22)	0.553	1.04 (0.89–1.21)	0.660
Fasting blood glucose < 100 mg/dL (n = 24,375)								
No CP	4261/4613 (92.4%)	18,220/19,762 (92.2%)	1		1		1	
CP ≥ 2	352/4613 (7.6%)	1542/19,762 (7.8%)	0.98 (0.87–1.10)	0.696	0.98 (0.87–1.11)	0.764	0.98 (0.87–1.11)	0.804
Fasting blood glucose ≥ 100 mg/dL (n = 19,595)								
No CP	3848/4181 (92%)	14,196/15,414 (92.1%)	1		1		1	
CP ≥ 2	333/4181 (8%)	1218/15,414 (7.9%)	1.01 (0.89–1.14)	0.894	1.02 (0.90–1.16)	0.766	1.00 (0.88–1.14)	0.991
Total cholesterol < 200mg/dL (n = 25,115)								
No CP	4769/5169 (92.3%)	18,368/19,946 (92.1%)	1		1		1	
CP ≥ 2	400/5169 (7.7%)	1578/19,946 (7.9%)	0.98 (0.87–1.09)	0.683	0.98 (0.88–1.10)	0.769	0.98 (0.87–1.10)	0.678
Total cholesterol ≥ 200mg/dL (n = 18,855)								
No CP	3340/3625 (92.1%)	14,048/15,230 (92.2%)	1		1		1	
CP ≥ 2	285/3625 (7.9%)	1182/15,230 (7.8%)	1.01 (0.89–1.16)	0.838	1.03 (0.90–1.18)	0.662	1.02 (0.89–1.17)	0.737
CCI score = 0 (n = 19,473)								
No CP	2458/2649 (92.8%	15,437/16,824 (91.8%)	1		1		1	
CP ≥ 2	191/2649 (7.2%)	1387/16,824 (8.2%)	1.01 (0.89–1.16)	0.838	1.03 (0.90–1.18)	0.662	0.85 (0.72–0.99)	0.040*
CCI score = 1 (n = 8885)								
No CP	1872/2030 (92.2%)	6335/6855 (92.4%)	1		1		1	
CP ≥ 2	158/2030 (7.8%)	520/6855 (7.6%)	1.03 (0.85–1.24)	0.767	1.01 (0.84–1.22)	0.931	1.01 (0.84–1.22)	0.897
CCI score ≥ 2 (n = 15,612)								
No CP	3779/4115 (91.8%)	10,644/11,497 (92.6%)	1		1		1	
CP ≥ 2	336/4115 (8.2%)	853/11,497 (7.4%)	1.11 (0.97–1.27)	0.122	1.11 (0.97–1.27)	0.121	1.11 (0.97–1.27)	0.129

Abbreviations: CP, chronic periodontitis; PD, Parkinson's disease; SBP, systolic blood pressure; DBP, diastolic blood pressure; CCI, Charlson Comorbidity Index.

* Conditional or unconditional logistic regression analysis, Significance at $p < 0.05$.

† Stratified model for age, sex, income, and region of residence.

‡ Model 1 was adjusted for smoking, alcohol consumption, obesity and CCI scores.

§ Model 2 was adjusted for model 1 plus total cholesterol, systolic blood pressure, diastolic blood pressure, and fasting blood glucose.

Supplementary Table S2. Crude and adjusted odd ratios of chronic periodontitis (CP) for Parkinson's disease (PD) when participants are diagnosed with CP ≥ 3 within 1 year before index date.

	N of PD	N of Control	Odd ratios for PD (95% confidence interval)					
	(exposure/total, %)	(exposure/total, %)	**Crude†**	*p*	**Model 1†‡**	*p*	**Model 2†§**	*p*
Total (n = 43,970)								
No CP	8395/8794 (95.5%)	33,671/35,176 (95.7%)	1		1		1	
CP ≥ 3	399/8794 (4.5%)	1505/35,176 (4.3%)	1.06 (0.95–1.19)	0.285	1.05 (0.94–1.18)	0.360	1.06 (0.95–1.19)	0.314
Age < 65 years old (n = 8380)								
No CP	1601/1676 (95.5%)	6378/6704 (95.1%)	1		1		1	
CP ≥ 3	75/1676 (4.5%)	326/6704 (4.9%)	0.92 (0.71–1.18)	0.506	0.91 (0.71–1.18)	0.495	0.90 (0.70–1.17)	0.452
Age ≥ 65 years old (n = 35,590)								
No CP	6794/7118 (95.5%)	27,293/28,472 (95.9%)	1		1		1	
CP ≥ 3	324/7118 (4.6%)	1179/28,472 (4.1%)	1.10 (0.97–1.25)	0.123	1.11 (0.98–1.26)	0.106	1.10 (0.97–1.25)	0.145
Men (n = 21,020)								
No CP	3980/4204 (94.7%)	15,987/16,816 (95.1%)	1		1		1	
CP ≥ 3	224/4204 (5.3%)	829/16,816 (4.9%)	1.09 (0.93–1.26)	0.290	1.09 (0.94–1.27)	0.256	1.09 (0.93–1.27)	0.275
Women (n = 22,950)								
No CP	4415/4590 (96.2%)	17,684/18,360 (96.3%)	1		1		1	
CP ≥ 3	175/4590 (3.8%)	676/18,360 (3.7%)	1.04 (0.88–1.23)	0.672	1.04 (0.88–1.23)	0.662	1.02 (0.86–1.21)	0.827
Low income (n = 18,740)								
No CP	3593/3748 (95.9%)	14,413/14,992 (96.1%)	1		1		1	
CP ≥ 3	155/3748 (4.1%)	579/14,992 (3.9%)	1.07 (0.90–1.29)	0.440	1.08 (0.90–1.30)	0.402	1.07 (0.89–1.29)	0.441
High income (n = 25,230)								
No CP	4802/5046 (95.2%)	19,258/20,184 (95.4%)	1		1		1	
CP ≥ 3	244/5046 (4.8%)	926/20,184 (4.6%)	1.06 (0.91–1.22)	0.454	1.06 (0.92–1.23)	0.430	1.04 (0.90–1.21)	0.559
Urban residents (n = 16,630)								
No CP	3139/3326 (94.4%)	12,601/13,304 (94.7%)	1		1		1	
CP ≥ 3	187/3326 (5.6%)	703/13,304 (5.3%)	1.07 (0.90–1.26)	0.438	1.07 (0.91–1.27)	0.411	1.06 (0.90–1.26)	0.480
Rural residents (n = 27,340)								
No CP	5256/5468 (96.1%)	21,070/21,872 (96.3%)	1		1		1	
CP ≥ 3	212/5468 (3.9%)	802/21,872 (3.7%)	1.06 (0.91–1.24)	0.462	1.06 (0.91–1.24)	0.437	1.05 (0.90–1.23)	0.545
Underweight (n = 1617)								
No CP	311/318 (97.8%)	1257/1299 (96.8%)	1		1		1	
CP ≥ 3	7/318 (2.2%)	42/1299 (3.2%)	0.67 (0.30–1.51)	0.340	0.71 (0.31–1.59)	0.402	0.74 (0.33–1.69)	0.479
Normal weight (n = 15,612)								
No CP	2944/3098 (95.0%)	12,003/12,514 (95.9%)	1		1		1	
CP ≥ 3	154/3098 (5.0%)	511/12,514 (4.1%)	1.23 (1.02–1.48)	0.029*	1.22 (1.01–1.47)	0.036*	1.21 (1.01–1.46)	0.044*
Overweight (n = 11,480)								
No CP	2202/2308 (95.4%)	8753/9172 (95.4%)	1		1		1	
CP ≥ 3	106/2308 (4.6%)	419/9172 (4.6%)	1.01 (0.81–1.25)	0.960	1.01 (0.81–1.26)	0.916	1.01 (0.81–1.26)	0.920
Obese (n = 15,261)								
No CP	2938/3070 (95.7%)	11,658/12,191 (95.6%)	1		1		1	
CP ≥ 3	132/3070 (4.3%)	533/12,191 (4.4%)	0.98 (0.81–1.19)	0.861	0.99 (0.82–1.21)	0.945	0.97 (0.79–1.18)	0.733
Non-smoker (n = 32,525)								
No CP	6478/6765 (95.8%)	24,731/25,760 (96%)	1		1		1	
CP ≥ 3	287/6765 (4.2%)	1029/25,760 (4.0%)	1.06 (0.93–1.22)	0.357	1.05 (0.92–1.21)	0.442	1.04 (0.91–1.19)	0.563
Past smoker and current smoker (n = 11,445)								
No CP	1917/2029 (94.5%)	8940/9416 (94.9%)	1		1		1	
CP ≥ 3	112/2029 (5.5%)	476/9416 (5.1%)	1.10 (0.89–1.36)	0.390	1.11 (0.89–1.37)	0.356	1.09 (0.88–1.36)	0.414
Alcohol consumption < 1 time a week (n = 29,626)								

No CP	2445/2551 (95.8%)	11,223/11,793 (95.2%)	1		1		1	
CP ≥ 3	106/2551 (4.2%)	570/11,793 (4.8%)	1.18 (1.03–1.35)	0.015*	1.17 (1.02–1.34)	0.024*	1.15 (1.01–1.32)	0.042*
Alcohol consumption ≥ 1 time a week (n = 14,344)								
No CP	5406/5669 (95.4%)	23,369/24,455 (95.6%)	1		1		1	
CP ≥ 3	263/5669 (4.6%)	1086/24,455 (4.4%)	0.85 (0.69–1.06)	0.143	0.87 (0.70–1.07)	0.189	0.87 (0.70–1.08)	0.197
SBP < 140 mmHg and DBP < 90 mmHg (n = 30,124)								
No CP	2989/3125 (95.7%)	10,302/10,721 (96.1%)	1		1		1	
CP ≥ 3	136/3125 (4.4%)	419/10,721 (3.9%)	1.05 (0.91–1.20)	0.510	1.05 (0.92–1.21)	0.452	1.05 (0.91–1.20)	0.524
SBP ≥ 140 mmHg or DBP ≥ 90 mmHg (n = 13,846)								
No CP	4409/4613 (95.6%)	18,947/19,762 (95.9%)	1		1		1	
CP ≥ 3	204/4613 (4.4%)	815/19,762 (4.1%)	1.12 (0.92–1.36)	0.266	1.09 (0.89–1.33)	0.387	1.07 (0.88–1.31)	0.488
Fasting blood glucose < 100 mg/dL (n = 24,375)								
No CP	4409/4613 (95.6%)	18,947/19,762 (95.9%)	1		1		1	
CP ≥ 3	204/4613 (4.4%)	815/19,762 (4.1%)	1.08 (0.92–1.26)	0.362	1.08 (0.92–1.27)	0.333	1.09 (0.93–1.27)	0.309
Fasting blood glucose ≥ 100 mg/dL (n = 19,595)								
No CP	3986/4181 (95.3%)	14,724/15,414 (95.5%)	1		1		1	
CP ≥ 3	195/4181 (4.7%)	690/15,414 (4.5%)	1.04 (0.89–1.23)	0.601	1.05 (0.89–1.24)	0.540	1.02 (0.87–1.21)	0.776
Total cholesterol < 200mg/dL (n = 25,115)								
No CP	4935/5169 (95.5%)	19,069/19,946 (95.6%)	1		1		1	
CP ≥ 3	234/5169 (4.5%)	877/19,946 (4.4%)	1.03 (0.89–1.20)	0.683	1.04 (0.90–1.21)	0.604	1.03 (0.89–1.20)	0.708
Total cholesterol ≥ 200mg/dL (n = 18,855)								
No CP	3460/3625 (95.5%)	14,602/15,230 (95.9%)	1		1		1	
CP ≥ 3	165/3625 (4.6%)	628/15,230 (4.1%)	1.11 (0.93–1.32)	0.248	1.12 (0.94–1.34)	0.204	1.11 (0.93–1.33)	0.251
CCI score = 0 (n = 19,473)								
No CP	2552/2649 (96.3%)	16,074/16,824 (95.5%)	1		1		1	
CP ≥ 3	97/2649 (3.7%)	750/16,824 (4.5%)	0.81 (0.66–1.01)	0.062	0.80 (0.65–1.00)	0.046*	0.81 (0.65–1.00)	0.049*
CCI score = 1 (n = 8885)								
No CP	1935/2030 (95.3%)	6582/6855 (96.0%)	1		1		1	
CP ≥ 3	95/2030 (4.7%)	273/6855 (4.0%)	1.18 (0.93–1.50)	0.167	1.17 (0.92–1.49)	0.203	1.17 (0.92–1.49)	0.207
CCI score ≥ 2 (n = 15,612)								
No CP	3908/4115 (95.0%)	11,015/11,497 (95.8%)	1		1		1	
CP ≥ 3	207/4115 (5.0%)	482/11,497 (4.2%)	1.21 (1.02–1.43)	0.025*	1.20 (1.02–1.42)	0.032*	1.20 (1.01–1.42)	0.034*

Abbreviations: CP, chronic periodontitis; PD, Parkinson's disease; SBP, systolic blood pressure; DBP, diastolic blood pressure; CCI, Charlson Comorbidity Index.

* Conditional or unconditional logistic regression analysis, Significance at $p < 0.05$.

† Stratified model for age, sex, income, and region of residence.

‡ Model 1 was adjusted for smoking, alcohol consumption, obesity and CCI scores.

§ Model 2 was adjusted for model 1 plus total cholesterol, systolic blood pressure, diastolic blood pressure, and fasting blood glucose.

Supplementary Table S3. Crude and adjusted odd ratios of chronic periodontitis (CP) for Parkinson's disease (PD) when participants are diagnosed with CP ≥ 1 within 2 years before index date.

	N of PD (exposure/total, %)	N of Control (exposure/total, %)	Odd ratios for PD (95% confidence interval)					
			**Crude†**	***p***	**Model 1†‡**	***p***	**Model 2†§**	***p***
Total (n = 43,970)								
No CP	6415/8794 (73.0%)	25,743/35,176 (73.2%)	1		1		1	
CP ≥ 1	2379/8794 (27.1%)	9433/35,176 (26.8%)	1.01 (0.96–1.07)	0.653	1.01 (0.96–1.07)	0.727	1.02 (0.96–1.07)	0.573
Age < 65 years old (n = 8380)								
No CP	1177/1676 (70.2%)	4660/6704 (69.5%)	1		1		1	
CP ≥ 1	499/1676 (29.8%)	2044/6704 (30.5%)	0.97 (0.86–1.09)	0.570	0.97 (0.86–1.09)	0.557	0.98 (0.87–1.10)	0.730
Age ≥ 65 years old (n = 35,590)								
No CP	5238/7118 (73.6%)	21,083/28,472 (74.1%)	1		1		1	
CP ≥ 1	1880/7118 (26.4%)	7389/28,472 (26.0%)	1.02 (0.97–1.09)	0.427	1.03 (0.97–1.10)	0.305	1.02 (0.97–1.09)	0.422
Men (n = 21,020)								
No CP	2983/4204 (71%)	11,984/16,816 (71.3%)	1		1		1	
CP ≥ 1	1221/4204 (29%)	4832/16,816 (28.7%)	1.02 (0.94–1.09)	0.692	1.03 (0.96–1.11)	0.431	1.03 (0.95–1.11)	0.478
Women (n = 22,950)								
No CP	3432/4590 (74.8%)	13,759/18,360 (74.9%)	1		1		1	
CP ≥ 1	1158/4590 (25.2%)	4601/18,360 (25.1%)	1.01 (0.94–1.09)	0.813	1.01 (0.93–1.09)	0.865	1.00 (0.93–1.08)	0.998
Low income (n = 18,740)								
No CP	2781/3748 (74.2%)	11,189/14,992 (74.6%)	1		1		1	
CP ≥ 1	967/3748 (25.8%)	3803/14,992 (25.4%)	1.02 (0.94–1.11)	0.585	1.03 (0.95–1.12)	0.508	1.02 (0.94–1.11)	0.608
High income (n = 25,230)								
No CP	3634/5046 (72.0%)	14,554/20,184 (72.1%)	1		1		1	
CP ≥ 1	1412/5046 (28.0%)	5630/20,184 (27.9%)	1.00 (0.94–1.08)	0.899	1.01 (0.94–1.08)	0.772	1.01 (0.94–1.08)	0.870
Urban residents (n = 16,630)								
No CP	2338/3326 (70.3%)	9301/13,304 (69.9%)	1		1		1	
CP ≥ 1	988/3326 (29.7%)	4003/13,304 (30.1%)	0.98 (0.90–1.07)	0.667	0.99 (0.91–1.08)	0.861	0.99 (0.91–1.08)	0.870
Rural residents (n = 27,340)								
No CP	4077/5468 (74.6%)	16,442/21,872 (75.2%)	1		1		1	
CP ≥ 1	1391/5468 (25.4%)	5430/21,872 (24.8%)	1.03 (0.97–1.11)	0.347	1.04 (0.97–1.11)	0.314	1.03 (0.96–1.10)	0.445
Underweight (n = 1617)								
No CP	237/318 (74.5%)	1023/1299 (78.8%)	1		1		1	
CP ≥ 1	81/318 (25.5%)	276/1299 (21.3%)	1.27 (0.95–1.68)	0.104	1.23 (0.92–1.65)	0.154	1.24 (0.92–1.66)	0.157
Normal weight (n = 15,612)								
No CP	2271/3098 (73.3%)	9262/12,514 (74.0%)	1		1		1	
CP ≥ 1	827/3098 (26.7%)	3252/12,514 (26.0%)	1.04 (0.95–1.13)	0.420	1.04 (0.95–1.14)	0.379	1.04 (0.95–1.14)	0.387
Overweight (n = 11,480)								
No CP	1669/2308 (72.3%)	6619/9172 (72.2%)	1		1		1	
CP ≥ 1	639/2308 (27.7%)	2553/9172 (27.8%)	0.99 (0.90–1.10)	0.887	1.00 (0.90–1.11)	0.973	1.01 (0.91–1.12)	0.868
Obese (n = 15,261)								
No CP	2238/3070 (72.9%)	8839/12,191 (72.5%)	1		1		1	
CP ≥ 1	832/3070 (27.1%)	3352/12,191 (27.5%)	0.98 (0.90–1.07)	0.662	0.99 (0.90–1.08)	0.782	0.97 (0.88–1.06)	0.476
Non-smoker (n = 32,525)								
No CP	4995/6765 (73.8%)	18,980/25,760 (73.7%)	1		1		1	
CP ≥ 1	1770/6765 (26.2%)	6780/25,760 (26.3%)	0.99 (0.93–1.05)	0.796	0.98 (0.93–1.05)	0.615	0.98 (0.92–1.04)	0.534
Past smoker and current smoker (n = 11,445)								
No CP	1420/2029 (70.0%)	6763/9416 (71.8%)	1		1		1	
CP ≥ 1	609/2029 (30.0%)	2653/9416 (28.2%)	1.09 (0.98–1.21)	0.096	1.12 (1.00–1.24)	0.044	1.11 (1.00–1.24)	0.053
Alcohol consumption < 1 time a week (n = 29,626)								

No CP	4617/6243 (74%)	17,454/23,383 (74.6%)	1		1		1	
CP ≥ 1	1626/6243 (26.1%)	5929/23,383 (25.4%)	1.04 (0.97–1.11)	0.265	1.03 (0.97–1.10)	0.367	1.02 (0.95–1.09)	0.611
Alcohol consumption ≥ 1 time a week (n = 14,344)								
No CP	1798/2551 (70.5%)	8289/11,793 (70.3%)	1		1		1	
CP ≥ 1	753/2551 (29.5%)	3504/11,793 (29.7%)	0.99 (0.90–1.09)	0.845	1.01 (0.92–1.11)	0.835	1.00 (0.91–1.10)	0.947
SBP < 140 mmHg and DBP < 90 mmHg (n = 30,124)								
No CP	4108/5669 (72.5%)	17,673/24,455 (72.3%)	1		1		1	
CP ≥ 1	1561/5669 (27.5%)	6782/24,455 (27.7%)	0.99 (0.93–1.06)	0.766	0.99 (0.93–1.06)	0.783	0.99 (0.92–1.05)	0.686
SBP ≥ 140 mmHg or DBP ≥ 90 mmHg (n = 13,846)								
No CP	2307/3125 (73.8%)	8070/10,721 (75.3%)	1		1		1	
CP ≥ 1	818/3125 (26.2%)	2651/10,721 (24.7%)	1.08 (0.99–1.18)	0.100	1.08 (0.99–1.19)	0.094	1.08 (0.98–1.18)	0.116
Fasting blood glucose < 100 mg/dL (n = 24,375)								
No CP	3376/4613 (73.2%)	14,499/19,762 (73.4%)	1		1		1	
CP ≥ 1	1237/4613 (26.8%)	5263/19,762 (26.6%)	1.01 (0.94–1.09)	0.799	1.02 (0.95–1.09)	0.633	1.02 (0.95–1.10)	0.556
Fasting blood glucose ≥ 100 mg/dL (n = 19,595)								
No CP	3039/4181 (72.7%)	11,244/15,414 (73%)	1		1		1	
CP ≥ 1	1142/4181 (27.3%)	4170/15,414 (27.1%)	1.01 (0.94–1.09)	0.736	1.02 (0.94–1.10)	0.675	1.00 (0.92–1.08)	0.994
Total cholesterol < 200mg/dL (n = 25,115)								
No CP	3738/5169 (72.3%)	14,579/19,946 (73.1%)	1		1		1	
CP ≥ 1	1431/5169 (27.7%)	5367/19,946 (26.9%)	1.04 (0.97–1.11)	0.260	1.04 (0.97–1.12)	0.252	1.04 (0.97–1.12)	0.268
Total cholesterol ≥ 200mg/dL (n = 18,855)								
No CP	2677/3625 (73.9%)	11,164/15,230 (73.3%)	1		1		1	
CP ≥ 1	948/3625 (26.2%)	4066/15,230 (26.7%)	0.97 (0.90–1.06)	0.506	0.98 (0.91–1.07)	0.697	0.97 (0.89–1.06)	0.513
CCI score = 0 (n = 19,473)								
No CP	1966/2649 (74.2%)	12,180/16,824 (72.4%)	1		1		1	
CP ≥ 1	683/2649 (25.8%)	4644/16,824 (27.6%)	0.91 (0.83–1.00)	0.051	0.90 (0.82–0.99)	0.024*	0.90 (0.82–0.99)	0.027*
CCI score = 1 (n = 8885)								
No CP	1486/2030 (73.2%)	5034/6855 (73.4%)	1		1		1	
CP ≥ 1	544/2030 (26.8%)	1821/6855 (26.6%)	1.01 (0.90–1.13)	0.834	1.00 (0.89–1.12)	0.976	1.00 (0.89–1.12)	0.980
CCI score ≥ 2 (n = 15,612)								
No CP	2963/4115 (72.0%)	8529/11,497 (74.2%)	1		1		1	
CP ≥ 1	1152/4115 (28.0%)	2968/11,497 (25.8%)	1.12 (1.03–1.21)	0.007*	1.11 (1.02–1.20)	0.012*	1.11 (1.02–1.20)	0.012*

Abbreviations: CP, chronic periodontitis; PD, Parkinson's disease; SBP, systolic blood pressure; DBP, diastolic blood pressure; CCI, Charlson Comorbidity Index.

* Conditional or unconditional logistic regression analysis, Significance at $p < 0.05$.

† Stratified model for age, sex, income, and region of residence.

‡ Model 1 was adjusted for smoking, alcohol consumption, obesity and CCI scores.

§ Model 2 was adjusted for model 1 plus total cholesterol, systolic blood pressure, diastolic blood pressure, and fasting blood glucose.
